# The crystal structure of TDP-43 RRM1-DNA complex reveals the specific recognition for UG- and TG-rich nucleic acids

**DOI:** 10.1093/nar/gkt1407

**Published:** 2014-01-23

**Authors:** Pan-Hsien Kuo, Chien-Hao Chiang, Yi-Ting Wang, Lyudmila G. Doudeva, Hanna S. Yuan

**Affiliations:** ^1^Institute of Molecular Biology, Academia Sinica, Taipei, Taiwan, ^2^Institute of Bioinformatics and Structural Biology, National Tsing Hua University, Hsin Chu, Taiwan and ^3^Graduate Institute of Biochemistry and Molecular Biology, National Taiwan University, Taipei 10048, Taiwan

## Abstract

TDP-43 is an important pathological protein that aggregates in the diseased neuronal cells and is linked to various neurodegenerative disorders. In normal cells, TDP-43 is primarily an RNA-binding protein; however, how the dimeric TDP-43 binds RNA via its two RNA recognition motifs, RRM1 and RRM2, is not clear. Here we report the crystal structure of human TDP-43 RRM1 in complex with a single-stranded DNA showing that RRM1 binds the nucleic acid extensively not only by the conserved β-sheet residues but also by the loop residues. Mutational and biochemical assays further reveal that both RRMs in TDP-43 dimers participate in binding of UG-rich RNA or TG-rich DNA with RRM1 playing a dominant role and RRM2 playing a supporting role. Moreover, RRM1 of the amyotrophic lateral sclerosis-linked mutant D169G binds DNA as efficiently as the wild type; nevertheless, it is more resistant to thermal denaturation, suggesting that the resistance to degradation is likely linked to TDP-43 proteinopathies. Taken together all the data, we suggest a model showing that the two RRMs in each protomer of TDP-43 homodimer work together in RNA binding and thus the dimeric TDP-43 recognizes long clusters of UG-rich RNA to achieve high affinity and specificity.

## INTRODUCTION

TDP-43 (TAR DNA-binding protein) is a DNA- and RNA-binding protein highly conserved in eukaryotes with multiple essential cellular functions in DNA transcription and RNA translation (1). Yet TDP-43 is abnormally aggregated forming inclusions in neuronal cells in amyotrophic lateral sclerosis (ALS) and frontotemporal lobar degeneration (FTLD) (2,3). TDP-43 inclusions have also been characterized in various neurodegenerative disorders, including Alzheimer's disease and other tauopathies and Lewy body disorders, suggesting a full spectrum of TDP-43 proteinopathies (4–6). Lines of evidence show that either the loss of TDP-43 normal cellular function or the gain of abnormal toxic function of TDP-43 inclusions may lead to neuronal cell death and disorders (7). To understand the normal cellular function of TDP-43, it is important to first elucidate how it selects and binds to its target RNA sequences for the regulation of various RNA metabolism events.

TDP-43 was first characterized as a DNA-binding protein bound to HIV-1 TAR DNA sequence for transcriptional repression (8). However, further studies have revealed that TDP-43 is primarily an RNA-binding protein with multiple roles in the regulation of mRNA splicing, translation and transportation, and it is also present in Drosha complex for micro RNA processing and in stress granules for protecting mRNAs in stress conditions (7,9). TDP-43 prefers to bind to UG-rich sequences of single-stranded RNA as demonstrated by its binding at the UG-repeats near exon 9 for splicing silence of the human *CFTR* gene, which encodes cystic fibrosis transmembrane conductance regulator and is linked to cystic fibrosis (10,11). The sequence preference of TDP-43 for binding at UG-repeats in the 3′ or 5′ splice sites of pre-mRNA transcripts, which promote exon skipping or inclusion, have also been observed in those of apolipoprotein AII (12), eukaryotic translation termination factor 1 (13), retinoid X receptor gamma (13), a breast cancer 1-mutated substrate (13) and polymerase delta interacting protein/S6 kinase 1 Aly/REF-like target (POLDIP3/SKAR) (14). Nevertheless, TDP-43 also binds to non-UG sequences as shown by its binding to the non-UG repeats in the 3′ UTR of its own mRNA for the autoregulation of TDP-43 levels (15). Genome-wide RNA mapping further confirmed that TDP-43 prefers to bind UG-rich sequences in a large set of RNA transcripts and has a broad role in the regulation of alternative splicing and gene expression (16–21). In these studies, beside UG repeats, TDP-43 also prefers to bind to poly(A)_n_ sequences (18), GC-rich sequences (18), a variant with an adenine in the middle (UG)_n_UA(UG)_m_ (17) and poly-pyrimidine rich sequences (16).

**Figure 1.**
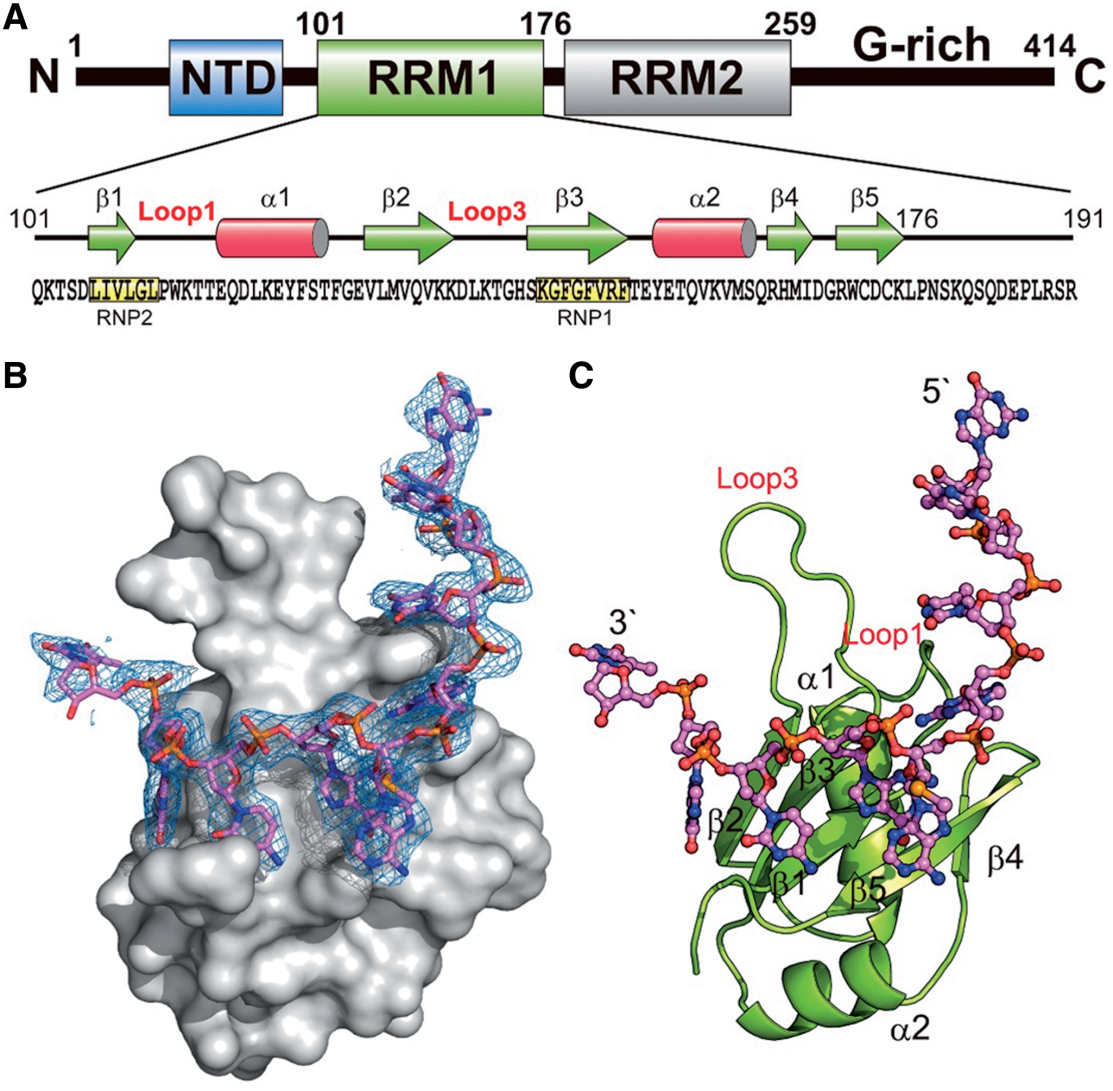
Overall crystal structure of hRRM1-DNA complex. (**A**) Domain structure of TDP-43. (**B**) The molecular surface of hRRM1 bound with a ssDNA. The difference Fourier (Fo-Fc) electron density map was superimposed on the structural model of DNA. (**C**) The overall crystal structure of hRRM1 in complex with DNA.

TDP-43 is actually well equipped for RNA binding, as it contains two tandem RNA recognition motifs RRM1 and RRM2, besides the N-terminal domain (NTD) and the C-terminal glycine-rich region (see Figure 1A). RRM, also known as RNA binding domain (RBD) and ribonucleoprotein domain (RNP), is one of the most abundant protein domains in eukaryotes (22). RRM contains two highly conserved segments denoted as RNP1 and RNP2 of eight and six amino acids, respectively. Typically, RRM has a fold of a β-sheet packed against two α-helices with the conserved aromatic/hydrophobic residues in RNP1 and RNP2 located in the β-sheet that stack with the bases and the sugar rings of the single-stranded RNA (23). Nevertheless, each RRM can interact with a minimum of two to a maximum of eight nucleotides and the interactions can be sequence specific or non-specific. Moreover, tandem repeats of RRM are frequently identified in RRM proteins and some of these proteins form oligomers, such as TDP-43 forming a homodimer with four RRMs (24–26). The interaction between RRMs and RNA is thus intricate, as each RRM may interact with RNA differently and tandem RRMs can be assembled in diverse ways (22).

The crystal structure of mouse RRM2 (mRRM2) in complex with a single-stranded DNA (ssDNA) has been reported revealing that ssDNA is indeed bound at the flat surface of the β-sheet (25). To establish the molecular basis for TDP-43 in binding RNA and DNA, here we report the crystal structure of human TDP-43 RRM1 in complex with a ssDNA. Together with mutagenesis and biochemical assays, we show that both RRM1 and RRM2 participate in nucleic acid binding to achieve its high affinity and preference in binding UG-rich RNA or TG-rich DNA. A structure model of TDP-43 homodimer binding to RNA further suggests how TDP-43 binds to mRNA transcripts with long UG-repeats.

## MATERIALS AND METHODS

### Protein expression and purification

The gene fragments encoding human TDP-43 RRM1 domain (residues 101–191) was amplified by polymerase chain reaction using the primers of 5′-GGGGGATCCCAGAAAACATCCGATTTA-3′ and 5′-GGGAAGCTTATTTCTGCTTCTCAAAGGCTC-3′. The polymerase chain reaction products were digested with BamHI and HindIII and then inserted into pQE30 expression vector (Qiagen) for the expression of the N-terminal His-tagged RRM1. The single-point mutants of hRRM1, W113A, T115A, F147L, F149L, D169G, D169A, R171A and N179A were generated by QuikChange® Site-Directed Mutagenesis Kit (Stratagene).

All of the TDP-43 RRM1 proteins were overexpressed in the *Escherichia coli* M15 strain. The cell culture (40 ml) was incubated overnight and then added into LB (1 l) medium with 50-mg ampicillin and allowed to grow to a density yielding an OD_600_ of ∼0.5 at 37°C. The cell culture was cooled down to 20°C and protein expression was induced by adding 0.8 mM IPTG and 50 mg of ampicillin for 22 h at 20°C.

The cells were lysed by microfluidizer and centrifuged at 12 000rpm for 30 min. Cell extracts were loaded onto a Ni-NTA affinity column (Qiagen) and the recombinant His-tagged protein was eluted by a step gradient using 50 mM phosphate buffer, 0.5 M imidazole, 100 mM NaCl and 10 mM β-mercaptoethanol at pH 7.5. Peak fractions were dialyzed against 50 mM phosphate buffer containing 50 mM imidazole and 10 mM β-mercaptoethanol at pH 7.5 and then applied to a SP column (GE Healthcare) and eluted with a step gradient of 50 mM phosphate, 1 M NaCl, 50 mM imidazole and 10 mM β-mercaptoethanol at pH 7.5. For further biochemical assays, the purified protein sample was dialyzed against a buffer solution of 50 mM phosphate (pH 7.5), 50 mM imidazole and 10 mM β-mercaptoethanol.

### Filter binding assay

The 30-nt (TG)_15_ DNA (10 pmol) were 5′-end labeled with [γ-^32^P]ATP by T4 PNK. The labeled DNA was then incubated with hRRM1 for 30 min at room temperature in the binding buffer of 50 mM phosphate, 50 mM imidazole and 10 mM β-mercaptoethanol at pH 7.5. The mixture was filtered through a BA 85 nitrocellulose membrane (Schleicher and Schuell) overlaid on a nylon membrane (Roche) in a 48-well slot blot apparatus (Bio-Rad). After extensive washing, the protein-DNA complex-bound nitrocellulose membrane and the DNA-bound nylon membrane were air dried and exposed to a ^Super^ RX-N Film (Fuji). The radioactive signals of labeled DNA were counted by UVP Biospectrum 600 Image System (UVP) and the affinity was calculated using the Logistic equation with three parameters. The dissociation constant *K*_d_ values were deduced from the protein concentrations at which half of the DNA substrates were bound with hRRM1.

### Circular dichroism

The thermal denaturing melting points of TDP-43 were measured three times by a circular dichroism (CD) spectrometer AVIV CD400. The CD spectra were scanned from 25 to 85°C at a wavelength of 208 nm and the melting point was estimated by AVIV program. The protein concentration was 0.3 mg/ml in a solution containing 100 mM NaCl in 50 mM phosphate buffer (pH 7.5).

### Crystallization, structure determination and refinement

The human RRM1 used for crystallization was purified with a procedure slightly different from the one used for biochemical analysis. All the Tris–HCl buffer was replaced by phosphate buffered saline buffer at pH 7.9, and 1 mM DTT was added in the last purification step using a HiTrap heparin column. The eluted proteins were dialyzed against 1% glycerol and 20 mM Tris–HCl at pH 7.9, and concentrated to ∼6 mg/ml by Vivaspin ultrafiltration unit (Sartorius). The purified hRRM1 was mixed with a Se-labeled ssDNA with a sequence of 5′-GTTGA_Se_GCGTT-3′ in a one-to-one molar ratio. This selenium-labeled DNA had a Se-H attached at the 2′-C of the sugar ring of adenine (SeNA Research Inc.). The crystals of hRRM1-DNA complex were grown by hanging drop vapor-diffusion method at room temperature, by mixing 1 μl of protein-DNA solution with 1 μl of reservoir solution containing 0.12 M CH_3_COONH_4_, 16% PEG 3350 and 0.05 M Bis-Tris at pH 5.5.

X-ray diffraction data were collected at SPXF beamline BL13C1 at NSRRC (Taiwan) at −150°C. The data were processed and scaled by HKL2000 (27) and all of the diffraction statistics are listed in Table 1. The RRM1-DNA complex crystallized in the P6_5_22 hexagonal space group, with one molecule per asymmetric unit. The structure of the complex was solved by single wavelength anomalous dispersion (SAD) and refined by PHENIX. The final model of hRRM1-DNA complex at 2.75 Å resolution contains 91 amino acids (residues 101–191), 9 nt (G1 to T9) and 14 water molecules. The data collection and refinement statistics are summarized in Table 1.

**Table 1.** Crystallographic statistics of hRRM1-DNA complex

	Values
Data collection and processing	
Wavelength (Å)	0.97622
Space group	P6_5_22
Cell dimensions (Å)	a = b = 71.11, c = 101.63
Resolution (Å)	2.75
Observed reflections	77 077
Unique reflections	4328
Redundancy^a^	17.8 (18.7)
Completeness^a^(%)	100 (99.9)
Rsym^a^	7.8 (55.4)
*I*/σ(*I*)^a^	43.5 (8.6)
Refinement statistics	
Resolution range	26.33–2.75
Reflections (work/test)	4301/430
R-factor/R-free (%)	20.65/25.86
Nonhydrogen atoms	
Protein	816
Solvent molecules	14
Model quality	
Root mean square deviations in	
Bond length (Å)	0.003
Bond angle (^o^)	0.75
Average B-factor (Å^2^)	54.99
Ramachandran plot (%)	
Most favored	98.67
Additionally allowed	1.33
Generously allowed	0
Disallowed	0

^a^The last shell (2.85–2.75A) statistics are listed in parenthesis.

## RESULTS

### Overall crystal structure of hRRM1-DNA complex

To reveal the molecular basis underlying the interactions between TDP-43 and nucleic acids, the N-terminal His-tagged human RRM1 (hRRM1, residues 101–191) was expressed in *E. coli* and purified by chromatographic methods for structural and biochemical studies. Various DNA and RNA with different sequences and lengths, including TG repeats and UG repeats, were used to co-crystallize with hRRM1, but only a single-stranded 10-nt DNA (5′-GTTGA_Se_GCGTT-3′) with a 2′-methylseleno-adenosine (A_Se_) could be co-crystallized with hRRM1. The hRRM1-DNA complex was crystallized in the hexagonal space group P6_5_22 with one hRRM1-DNA per asymmetric unit by the hanging-drop vapor diffusion method. The structure of the hRRM1-DNA complex was solved by SAD using X-ray diffraction data collected at Se-absorption edge by PHENIX. After auto-build of the protein peptide chain, the Fourier map (2Fo-Fc) revealed a clear continuous electron density, which could be fitted with 9 nt from G1 to T9 (See Figure 1B). The final refined model contained one hRRM1 polypeptide chain (residues 103–179) and one ssDNA (G1 to T9) with an R-factor/R-free of 20.65/25.86% for 4301/430 reflections up to a resolution of 2.75 Å.

The overall crystal structure of the hRRM1-DNA complex showed that hRRM1 had an αβ sandwich structure containing a five-stranded β-sheet packed against two α-helices (see Figure 1C). Similar to the mRRM2, the five-stranded β-sheet in hRRM1 had a topology of β2-β3-β1-β5-β4 with the conserved RNP2 and RNP1 segments located in β1 and β3, respectively. However, different from mRRM2, hRRM1 had a longer loop between β2 and β3, named Loop3. The ssDNA was bound on the flat surface of the β-sheet of hRRM1 interacting mainly with RNP2 and RNP1 segments. The 5′-end of the DNA extended upward and further interacted with the Loop1 residues.

### Interactions between hRRM1 and DNA

**Figure 2.**
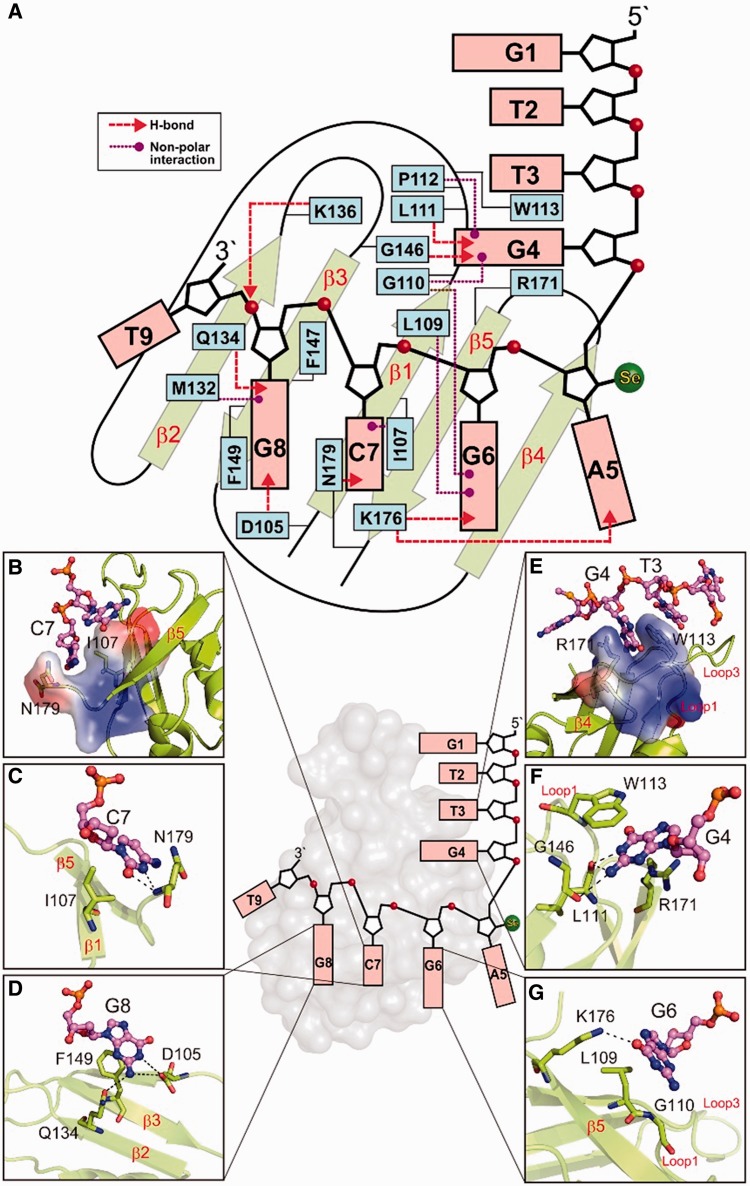
The interactions between hRRM1 and DNA. (**A**) Schematic diagram of the interactions between hRRM1 and DNA. I107, F147 and F149 are the conserved aromatic/hydrophobic residues in RNP2 and RNP1 segments that interact with DNA bases and sugar rings of C7-G8. (**B**) The cytosine of C7 stacks with the side chains of I107 and N179. (**C**) C7 forms hydrogen bonds to N179. (**D**) G8 forms hydrogen bonds with D105 and Q134. (**E**) G4 stacks between R179 and W113, whereas T3 stacks with W113. (**F**) G4 hydrogen bonds with L111, W113 and G146. (**G**) G6 hydrogen bonds with K176.

The ssDNA bound primarily on the surface of the central β-sheet and the schematic diagram of the detailed interactions between hRRM1 and DNA is shown in Figure 2A. The 3′-end nucleotides, C7-G8, interacted most extensively with the RNP segments in the β-sheet. Similar to the classical RRM proteins, the conserved I107 in RNP2 formed nonbonded interactions with C7 and the conserved F149 in RNP1 stacked with G8 (see Figure 2A and B). The cytosine of C7 inserted into in a cleft on the hRRM1 surface, not only stacking with I107 but also forming hydrogen bonds with N179 (see Figure 2C). The aromatic side chain of the conserved F147 in RNP1 was inserted between the two sugar rings of C7 and G8. Besides stacking with F149, the G8 base also formed hydrogen bonds to hRRM1: N1 and N2 formed hydrogen bonds with residue D105 (Oδ2); N2 formed a hydrogen bond with Q134 (Oε1) (Figure 2D). The G6 and A5 nucleotides did not stack with any amino acid residues but they formed hydrogen bonds with hRRM1: the O6 atom of G6 formed a hydrogen bond with K176 (Nζ), and the N1 atom of A5_Se_ formed a hydrogen bond with K176 (Nζ) (Figure 2G). The extensive hydrogen-bonding networks surrounding G6, C7 and G8 likely specified the sequence preference for G-C-G at these three binding sites on the central β-sheet.

The T3 and G4 nucleotides interacted extensively with Loop1 of hRRM1. G4 inserted into the surface cleft, stacking with W113 (in Loop 1) and R171, and formed extensive hydrogen bonds with hRRM1: N1 and N2 to L111 (backbone O); O6 to W113 (backbone N); N2 to G146 (backbone O); N7 to R171 (Nη1) (see Figure 2E and F). Moreover, T3 also stacked with W113 from Loop1, but this π-π stacking interaction probably cannot specify the preference for T3 at this position. In contrast, the extensive hydrogen bond network surrounding G4 might specify the sequence preference for a G at this binding site. The long loop, Loop3, interacted with the DNA phosphate backbone of G8, suggesting that it might be crucial for the recognition of the shape but not the sequence of the ssDNA. In summary, hRRM1 bound the ssDNA via nonbonded, π-π stacking interactions and hydrogen bonding, and it might specify the preference for a GC-rich sequence: X-G4-X-G6-C7-G8.

### Mutation of the critical residues for nucleic acid binding and TDP-43 pathology in RRM1

**Figure 3.**
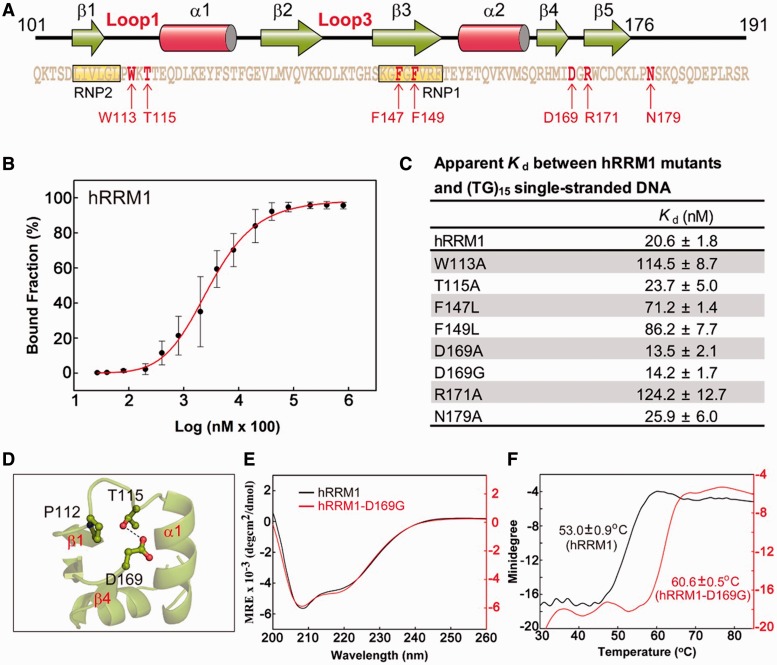
Mutation of the critical residues for nucleic acid binding and TDP-43 pathogenesis in hRRM1. (**A**) Mutation of a number of residues (indicated by arrows) in hRRM1. (**B**) The binding affinity between the wild-type hRRM1 and (TG)_15_ was measured by the nitrocellulose filter binding assay. The 5′-end ^32^P-labeled DNA (10 pmol) was incubated with hRRM1 (0.0002–300 µM) and the hRRM1-DNA complexes trapped in the nitrocellulose filters were quantified. (**C**) The apparent *K*_d_ between hRRM1 mutants and (TG)_15_. (**D**) D169 located in Loop6 between β4 and β5 forms a hydrogen bond to the side chain of T115 located in Loop1. (**E**) The CD spectra of hRRM1 and hRRM1-D169G mutant. (**F**) The thermal melting points of hRRM1 and hRRM1-D169G mutant were estimated by CD at a wavelength of 208 nm.

To verify that the interactions observed between hRRM1 and DNA in the crystal structure were critical for nucleic acid binding in physiological conditions in solutions, we further constructed several single-point mutants in hRRM1, including W113A, T115A, F147L, F149L, D169G, D169A, R171A and N179A (Figure 3A). Five of the mutated residues participated in hRRM1-DNA interactions in the crystal structure: W113, R171 and N179 in loop regions, and F147 and F149 in the RNP1 segment. Moreover, we also constructed the D169G mutant because mutation of D169 to glycine in TDP-43 was reported to associate to sporadic ALS (28). D169 formed a hydrogen bond to T115, and therefore T115A, as well as D169A, were also constructed for the comparison of its biochemical properties with those of D169G (see Figure 3D).

The seven single-point His-tagged hRRM1 mutants were expressed and purified and their DNA-binding affinities to a 30-nt ssDNA with tandem TG repeats [(TG)_15_] were measured by nitrocellulose filter binding assays. The estimated dissociation constant (*K*_d_) for the wild-type hRRM1 against (TG)_15_ was 20.6 ± 1.8 nM (Figures 3B and C). The two loop mutants, W113A and R171A, had a 6-fold higher *K*_d_ of 114.5 ± 8.7 and 124.2 ± 12.7 nM, respectively. The two RNP1 mutants, F147L and F149L, had a 3- to 4-fold higher *K*_d_ of 71.2 ± 1.4 and 86.2 ± 7.7 nM, respectively. However, the N179A mutant only had a slightly higher *K*_d_ of 25.9 ± 6.0 nM than that of the wild-type hRRM1. These results confirm that the interface residues observed in the crystal structure indeed contribute to the interactions between hRRM1 and DNA in the low salt buffer at pH 7.5.

The disease-related mutant D169G unexpectedly had a slightly lower *K*_d_ of 14.2 ± 1.7 nM than that of the wild-type hRRM1 (20.6 ± 1.8 nM). Similarly, D169A also had a slightly lower *K*_d_ of 13.5 ± 2.1 nM, suggesting that mutation at D169 to G or A could not impair the DNA binding activity of hRRM1. Moreover, T115A also had a retained *K*_d_ of 23.7 ± 5.0 nM. Apart from the RRM1 D169G mutant, we also constructed a D169G hN12 mutant using the C-terminal tail-truncated hTDP-43 (hN12, residues 1–259) as the template. We found that this D169G hN12 mutant bound RNA [(UG)_15_] with a similar affinity (*K*_d_ = 5.5 ± 0.5 nM) as the wild-type hN12 (*K*_d_ = 5.3 ± 1.2 nM) (Figure 5A). This result further showed that D169G mutant retained its ability in RNA binding.

Further analysis by CD showed that hRRM1 D169G had a similar CD spectrum as compared with that of the wild-type hRRM1, suggesting that D169G had a retained folded structure. It was thus intriguing why D169G bound DNA slightly better than wild-type RRM1 because D169 was not involved in the interactions between hRRM1 and DNA. A further assay showed that D169G in fact had a higher thermal stability with a melting point of 60.6°C as compared with the 53.0°C melting point of wild-type hRRM1 as monitored by CD at 208 nm (Figure 3E). Thus a single-point mutation of D169 to G had a profound effect that the melting point of hRRM1 was increased by ∼7°C. In summary, D169G hRRM1 mutant had a retained overall structure with increased thermal stability and bound single-stranded TG-repeats with a slightly higher affinity as compared with the wild-type hRRM1.

### RRM1 interacts more extensively with nucleic acids than RRM2

**Figure 4.**
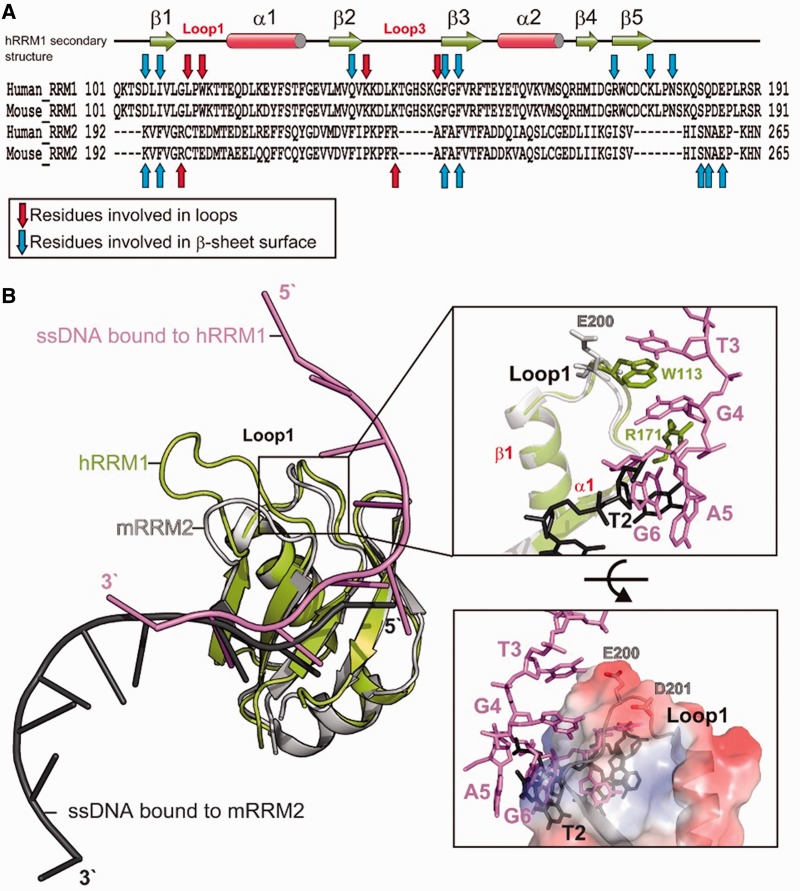
hRRM1 interacts more extensively with DNA, as revealed by the comparison between the crystal structures of hRRM1-DNA and mRRM2-DNA. (**A**) Sequence alignment of human and mouse RRM domains. Blue and red arrows mark the residues that interact with ssDNA via nonbonded interactions or hydrogen bonding. Red arrows are located in the loops and blue arrows are located within or close to β-strands. (**B**) The crystal structure of hRRM1-DNA (green and purple) is superimposed on mRRM2-DNA (gray and black) complex. A close look in the right top panel shows that only Loop1 of hRRM1 interacts with DNA: W113/R171 stacking with T3/G4. On the other hand, the Loop1 of RRM2 does not interact with DNA because the molecular surface of Loop1 is acidic and not suitable for DNA binding as shown in the right bottom panel.

The two RRMs in hTDP-43 share a high sequence identity of 22%; however, hRRM1 is longer than hRRM2 due to longer loops (see Figure 4A). The crystal structure of mRRM2 in complex with the ssDNA was reported (PDB: 3D2W) that had an identical sequence to the one in the hRRM1-DNA complex. The comparison of the protein-nucleic acid interactions between hRRM1 and mRRM2 showed that most of the residues that interacted with nucleic acids were located within or next to the β-sheet (blue arrows) and the rest of interacting residues were located in the Loop1 and Loop3 regions (red arrows in Figure 4A). The hRRM1 interacted more extensively with ssDNA than mRRM2 (9 versus 12 arrows) because more residues in hRRM1 participated in the interactions. These results are consistent with the previous binding assays showing that RRM1 plays a more critical role in the nucleic acid binding than RRM2 (10).

A close examination between the two complex structures revealed that the β-sheet residues in mRRM2 mainly interacted with T3-G4, and in contrast, the β-sheet residues in hRRM1 interacted with C7-G8. Moreover, hRRM1 had extra interactions with T3-G4 via its loop residues of L111 and W113. The stacking between T3/W113/G4/R171 was only observed in the hRRM1-DNA complex because Loop1 in mRRM2 was located more distantly from the DNA and the aligned E200 and I253 in the same position either pointed away or was located too far away from DNA (Figure 4B). The molecular surface of Loop1 of mRRM2 clearly showed an acidic surface that is not suitable for binding RNA/DNA (bottom panel in Figure 4B). On the other hand, the mutations of W113 and R171 to alanine generated defective hRRM1 mutants in DNA binding, suggesting that the interactions around RRM1 Loop1 region were critical for the DNA binding activity of TDP-43. In summary, our results confirm that RRM1 of TDP-43 interacts more extensively with nucleic acids and that RRM1 prefers binding to G4-X-G6-C7-G8 sequence, whereas RRM2 prefers binding to T3-G4 sequence.

### TDP-43 binds TG-rich DNA and UG-rich RNA with a high affinity

To determine how TDP-43 binds DNA and RNA via its RRMs, a number of human TDP-43 mutants were constructed using the C-terminal tail-truncated hTDP-43 (hN12, residues 1–259) as the template that had the NTD, RRM1 and RRM2, but not the C-terminal tail. Single- and double-point mutants in RRM1 were constructed with mutations in the RNP2 segment, including hN12-F147A, hN12-F149A and hN12-F147A-F149A. Similarly, single- and double-point mutants in RRM2 in the aligned RNP2 segment were constructed, including hN12-F229A, hN12-F231A and hN12-F229A-F231A. The DNA-binding and RNA-binding affinities of these mutants to (TG)_15_ and (UG)_15_ were measured by nitrocellulose filter binding assays.

**Figure 5.**
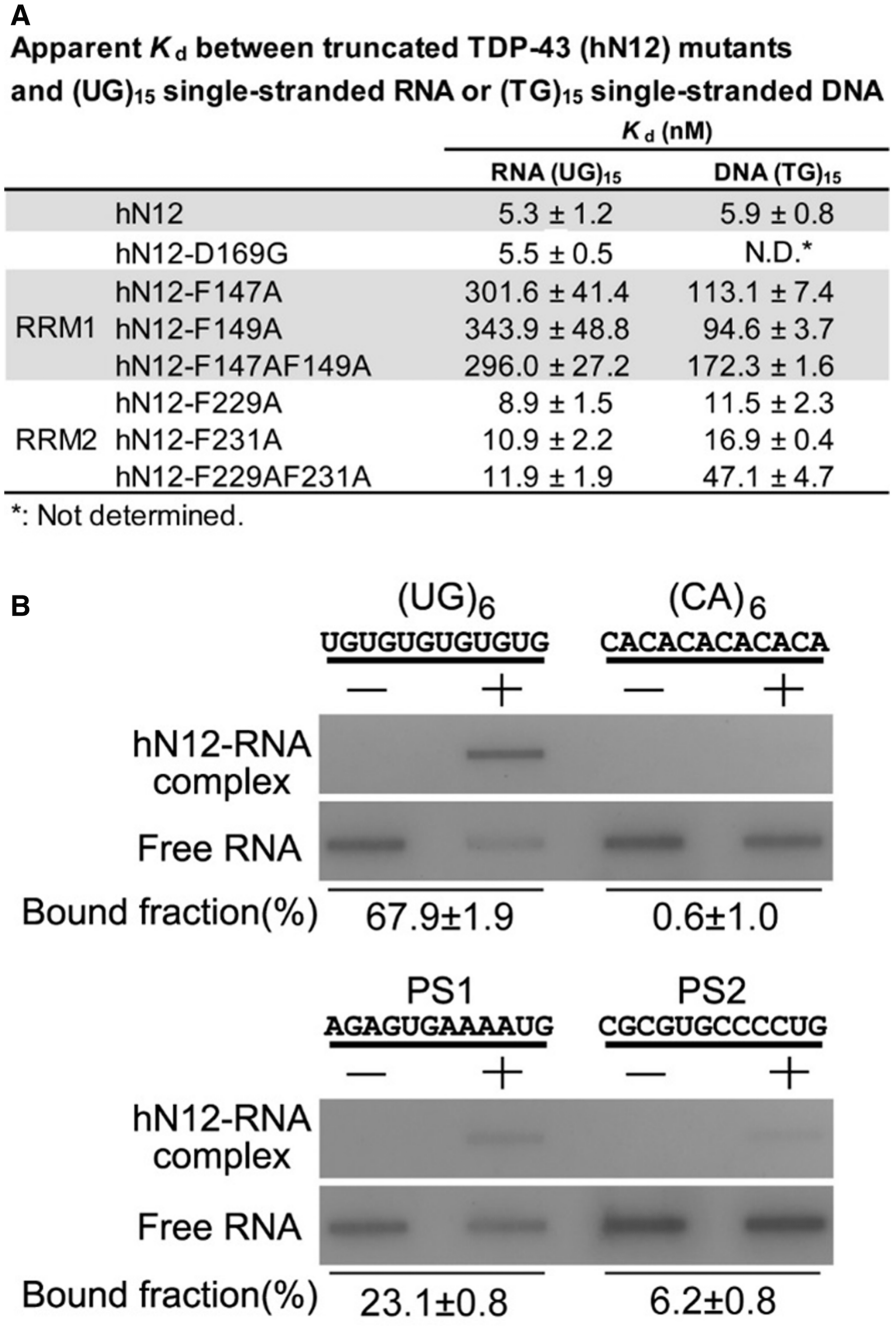
Mutations in either RRM1 or RRM2 reduce the TDP-43 high-affinity binding for UG-repeated RNA or TG-repeated DNA. (**A**) The C-terminal truncated TDP-43 (hN12, residues 1–259) was incubated with 5′-end ^32^P-labeled single-stranded (UG)_15_ RNA or (TG)_15_ DNA (10 pmol) for the measurement of binding affinity by the nitrocellulose filter binding assay. The estimated apparent *K*_d_ between the wild-type and mutated hN12 show that mutations in both RRM1and RRM2 generated defective mutants in RNA and DNA binding. (**B**) The nitrocellulose filter binding assays reveal sequence specificity of TDP-43 for UG repeats and UG-rich sequences. The hN12 (2 µM) bound to (UG)_6_ with the highest affinity (estimated bound fraction: 67.9 ± 1.9%), the UG-rich PS1 and PS2 with moderate affinity (23.1 ± 0.8 and 6.2 ± 0.8%) and (CA)_6_ with the lowest affinity (0.6 ± 1.0%).

The wild-type hN12 bound tightly to both single-stranded RNA and DNA with a *K*_d_ of 5.3 ± 1.2 nM for (UG)_15_ and 5.9 ± 0.8 nM for (TG)_15_ (Figure 5A). Mutations in RRM1 largely reduced the binding affinity by ∼50-fold for UG-repeated RNA and ∼20-fold for TG-repeated DNA, suggesting that the mutated phenylalanine residues play a major role in both RNA and DNA binding in TDP-43. Mutations in RRM2 also reduced the binding affinity but only ∼2-fold for UG-repeated RNA and 2- to 8-fold for TG-repeated DNA. These results suggest that RRM1 of hTDP-43 plays a more dominant role in nucleic acid binding, whereas RRM2 also participates in the interactions. Moreover, this result suggests that TDP-43 likely binds to single-stranded DNA and RNA in a similar binding mode and therefore mutations in TDP-43 produces similar effects of reduced binding for DNA and RNA.

To further confirm the specific interactions for UG-rich sequences, different sequences of 12-nt RNA were synthesized for binding assays, including a UG repeat (UG)_6_, two UG-rich putative sites PS1 (AGAGUGAAAAUG) and PS2 (CGCGUGCCCCUG) and a putative low-affinity sequence (CA)_6_. TDP-43 N12 bound to (UG)_6_ with the highest affinity (estimated bound fraction: 67.9 ± 1.9%) and bound to PS1 (23.1 ± 0.8%) and PS2 (6.2 ± 0.8%) with moderate affinity, whereas it did not bind to (CA)_6_ (0.6 ± 1.0%) (see Figure 5B). This result suggests that TDP-43 indeed has specificity for UG repeats and UG-rich sequences.

## DISCUSSION

### How TDP-43 binds RNA with tandem UG repeats

RNA-recognition motif (RRM) is the most abundant RBD in higher vertebrates with an estimation that RRMs are present in 0.5–1% of human gene products (29). RRM-containing proteins participate in various posttranscriptional RNA processing, such as mRNA splicing, editing, export, stability regulation and turnover (30). Most of the eukaryotic RRM-containing proteins bear multiple copies of RRM so that they can bind a long stretch of RNA with high affinity and/or sequence specificity, with examples including hnRNPA1 (2 RRMs), Sex-lethal (2 RRMs), FIR (2 RRMs), Prp24 (3 RRMs), PABP (4 RRMs) and PTB (4 RRMs) (31–36). Moreover, RRM proteins often form homodimers, and these dimers contain double the copies of RRMs for RNA binding. All of the RRM structures reported to date show that RRM binds single-stranded RNA or DNA on the β-sheet surface interacting primarily with the conserved aromatic/hydrophobic residues in RNP1 and RNP2 segments.

Similar to other RRM proteins, TDP-43 contains multiple copies of RRMs and forms a homodimer, and as a result, the dimeric TDP-43 has four copies of RRMs. It has been suggested that TDP-43 forms a dimer through the interactions between its NTDs (24,26,37). Our binding assays show that the dimeric C-terminal truncated TDP-43 (N12, residues 1–259) binds single-stranded (UG)_15_ repeats with a high affinity and an estimated dissociation constant of 5.3 nM. Our mutational results further reveal that the binding affinity between TDP-43 (N12) and ssRNA is greatly reduced by mutations in RRM1 and moderately reduced by mutations in RRM2, suggesting that RRM1 plays a more dominant role and that RRM2 also participates in the interactions but plays only a supporting role in RNA binding. This binding result is consistent with the crystal structures showing that RRM1 interacts more extensively with the bound DNA. The high binding affinity between TDP-43 and (UG)_15_ could be due to the contribution from the multiple copies of RRMs.

**Figure 6.**
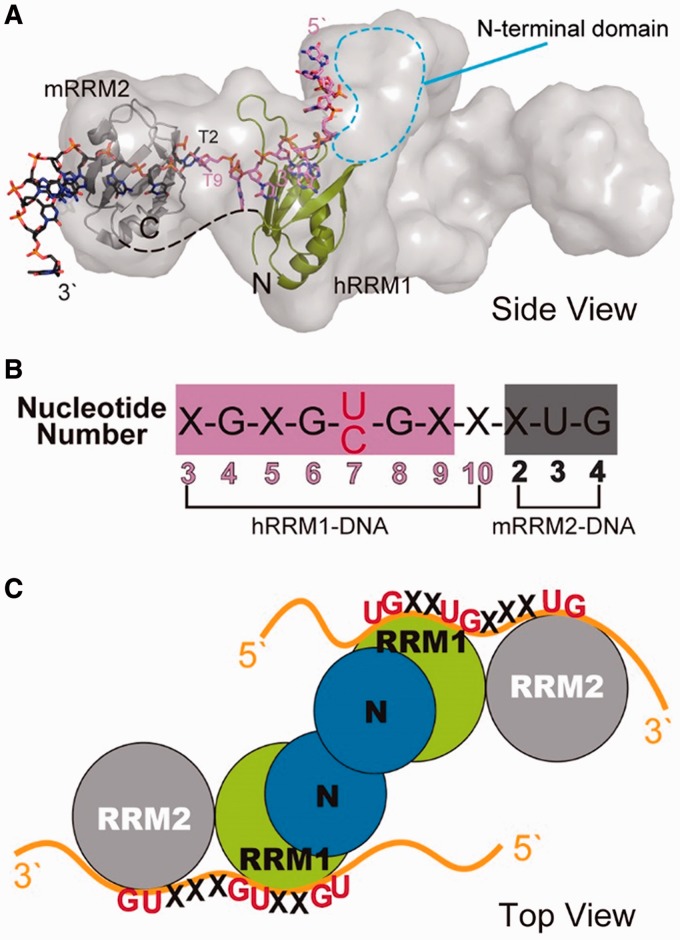
The structural model of TDP-43 bound to single-stranded nucleic acids. (**A**) The SAXS envelope of TDP-43 dimer fitted with the crystal structure of hRRM1-DNA and mRRM2-DNA in the orientation that the DNA forms a continuous 5′–3′ strand, as it is bound from hRRM1 to mRRM2 in TDP-43. (**B**) The putative RNA binding sequence of TDP-43 is derived from the sequence in hRRM1-DNA and mRRM2-DNA complexes. (**C**) TDP-43 homodimer likely binds to a long UG-rich RNA via its RRM1 and RRM2 domains.

How does TDP-43 bind long clusters of UG-rich sequences in pre-mRNA transcripts? Previously, we determined the low-resolution structure of TDP-43 N12 by the small angle X-ray scattering (SAXS) (37). The SAXS structure envelope clearly reveals an elongated conformation and one half of the envelope can be fitted with three domains: NTD, RRM1 and RRM2 (see Figure 6A). Previous studies reveal that TDP-43 forms a homodimer via its NTD, and therefore NTDs are suggested to locate in the middle with two RRM2 domains flanking outward at the two sides of the elongated envelope. It is difficult to predict how RRM1 and RRM2 are assembled in TDP-43 because RRM can be associated with each other in diverse ways (22). However, we found that RRM1 and RRM2 can be oriented in a way in the SAXS envelope that the two bound ssDNAs can form a continuous 5′–3′ strand as it extends from the β-sheet surface of RRM1 to that of RRM2 (Figure 6).

Several *in vitro* studies showed that TDP-43 preferentially binds the TG/UG dinucleotide repeat element (10,17,18,38). In the crystal structure of hRRM1-DNA complex, Loop1 residues recognize the T3-G4 dinucleotide, whereas the β-sheet interacts with G6-C7-G8. The C7 can be replaced by T7 that can also fit snugly and form a hydrogen bond with N179. Therefore, it appears that RRM1 can specifically recognize RNA with a sequence of X-G-X-G-U/C-G (see Figure 6B). On the other hand, mRRM2 only recognizes a T3-G4 via the β-sheet residues. Based on the model of TDP-43-RNA complex, we thus generate the RNA sequence that is likely recognized by TDP-43: X-G-X-G-U/C-G-X-X-X-U-G (see Figure 6B). This putative sequence showed that it is not only limited to UG repeats but can accommodate some other sequences in between the UG sequences, consistent with the TDP-43 binding sites that are not only limited but rich in UG repeats.

Interestingly, the solution structure of TDP-43 RRM1-RRM2 (residues 102–269) in complex with a single-stranded RNA has been reported recently showing a similar arrangement of RRM1 and RRM2 bound to the extended RNA from 5′ to 3′ end, as that in our model (39). The interactions between TDP-43 RRM1-RRM2 and RNA also share similar sequence specificity as we observed in the crystal structures with the RRM1 specifically recognizing G1-N-G3-U4-G5 and RRM2 recognizing U8-G9 in the 12-nt ssRNA with a sequence of 5′-G1-U2-G3-U4-G5-A6-A7-U8-G9-A10-A11-U12-3′. The similar specific interactions observed in the NMR solution and X-ray crystal structures suggest that TDP-43 has a preference and similar binding mode for the UG-rich and TG-rich sequences.

It remains unclear if all of the four RRMs interact with one strand or separate strands of RNA, or only the two RRM1 in TDP-43 dominate the interactions. Our previous data and other studies reported that at least 6 UG repeats were required for TDP-43 high-affinity binding and longer UG repeats were bound by TDP-43 with higher affinities (10,25). The cross-linking-immunoprecipitation sequencing studies also revealed long UG-rich sequences of >100 nt (16,19,20). Taken together this suggests that TDP-43 not only binds UG repeats via its RRM1 and RRM2, and both protomers might interact with a long stretch of RNA to increase the binding affinity and specificity (see the model in Figure 6C). It is also possible that the long RNA with multiple binding regions accommodates more than one TDP-43 for interactions.

### Disease-related mutation of D169G in TDP-43

Numerous mutations have been identified in TDP-43 that are linked to ALS and FTLD (6). These mutations are mostly located in the C-terminal glycine-rich tail with the exception of D169G, which is the only mutation located in RRM1 (28). In the crystal structure of hRRM1-DNA complex, D169 is located in Loop6 between β4 and β5, and it hydrogen bonds to the side chain of T115 located in Loop1 between β1 and α1 (Figure 3C). This interaction thus appears critical and may stabilize the structure of Loop1 and Loop6. However, unexpectedly, we found that D169G hRRM1 mutant had a retained overall structure and binds single-stranded (TG)_15_ with a slightly higher affinity than the wild-type hRRM1. These results suggest that the disease-linked D169G mutation in TDP-43 did not produce any defect in protein folding or RNA binding.

The only significant biochemical phenotype of D169G we observed here is that it was more resistant to thermal denaturation with a melting temperature of ∼7°C higher than that of wild-type hRRM1 as monitored by CD. The unique features of TDP-43 proteinopathies include its mislocation and aggregation in cytoplasm. The mislocated cytoplasmic TDP-43 can be degraded through the ubiquitin-proteasome and autophagosome-mediated degradation systems (40). It has been shown that D169G had a reduced binding and a decreased co-aggregation with UBQLN, suggesting that D169G mutant is degraded by the proteasome less efficiently (41). Alternatively, it has been shown that D169 is located in one of the potential caspase cleavage sites (DXXD) of TDP-43, and D169G is a cleavage-resistant mutant. The mutation of D169G thus results in a higher level of the full-length TDP-43 with stronger death-inducing activity in the cytoplasm (42).

Here we further provide a solid line of evidence showing that D169G hRRM1 mutant is more thermal stable than the wild-type hRRM1. Hence, D169G mutant is probably not only more resistant to proteasome digestion and caspase cleavage, but it is more stable with a longer half-life than that of wild-type TDP-43. It has been shown that ALS-linked mutations exhibit longer protein half-lives, about 12 h for wild-type TDP-43 and 24–48 h for ALS-linked mutants (43). Therefore, it is likely that the increased thermal stability of D169G is correlated to its resistance to degradation and a higher level of TDP-43 for eliciting TDP-43 proteinopathies.

## CONCLUSION

This study reveals how the RRM1 domain of TDP-43 interacts extensively with the ssDNA via the β-sheet and loop residues at atomic level. Taken together the structural, biochemical and mutational results, we conclude that both RRMs in TDP-43 participate in binding of a long stretch of nucleic acids with RRM1 playing a dominate role and RRM2 playing a supporting role. TDP-43 thus binds long clusters of UG-rich RNA via its four RRM domains in the two protomers to achieve high affinity and specificity.

## ACCESSION NUMBERS

4IUF.
